# Rapid detection of infectious bovine Rhinotracheitis virus using recombinase polymerase amplification assays

**DOI:** 10.1186/s12917-017-1284-0

**Published:** 2017-12-13

**Authors:** Peili Hou, Hongmei Wang, Guimin Zhao, Chengqiang He, Hongbin He

**Affiliations:** Key Laboratory of Animal Resistant Biology of Shandong, Ruminant Diseases Research Center, College of Life Sciences, Shandong Normal University, No. 88 East Wenhua Road, Jinan City, Shandong Province China

**Keywords:** Infectious bovine rhinotracheitis virus (IBRV), Recombinase polymerase amplification (RPA), Lateral-flow dipstrip (LFD), Rapid detection

## Abstract

**Background:**

Infectious bovine rhinotracheitis virus (IBRV) is a major pathogen in cattle and has led to significant economic losses to the dairy industry worldwide, and therefore a more optimal method for the rapid diagnosis of IBRV infection is highly needed. In this study, we described the development of a lateral flow dipstrip (LFD) of isothermal recombinase polymerase amplification (RPA) method for rapid detection of IBRV.

**Methods:**

Distinct regions were selected as a candidate target for designing the LFD-RPA primers and probes. The analytical sensitivity of the RPA assay was determined using ten-fold serially diluted IBRV DNA. The specificity of the assay was assessed with other viral pathogens of cattle with similar clinic and other herpesviruses. The clinical performance was evaluated by testing 106 acute-phase high fever clinical specimens.

**Results:**

RPA primers and probe were designed to target the specific conserved UL52 region fragment of IBRV. The detection could be completed at a constant temperature of 38 °C for 25 min, and the amplification products were easily visualized on a simple LFD. The detection limit of this assay was 5 copies per reaction of IBRV DNA and there was no cross-reactivity with other viruses causing bovine gastrointestinal and respiratory infections or other herpesviruses. The assay performance on acute-phase high fever clinical samples collected from cattle with no vaccine against IBRV, which were suspected to be infected with IBRV, was validated by detecting 24 fecal, 36 blood, 38 nasal swab and 8 tissue specimens, and compared with SYBR Green I based real-time PCR. The coincidence between IBRV LFD-RPA and real-time PCR was 100%.

**Conclusion:**

IBRV LFD-RPA was fast and much easier to serve as an alternative to the common measures used for IBRV diagnosis, as there is reduction in the use of instruments for identification of the infected animals. In addition, this assay may be the potential candidate to be used as point-of-care diagnostics in the field.

## Background

Infectious bovine rhinotracheitis virus (IBRV), often referred to as bovine herpesvirus 1 (BoHV-1), is responsible for infectious bovine rhinotracheitis (IBR). IBR is highly contagious, and can cause a diverse range of clinical manifestations from upper respiratory disease, rhinitis, vulvovaginitis, traeheitis, enteritis, conjunctivitis, abortion and encephalitis, to fatal systemic infection in neonatal calves [1]. IBRV can also induce immune suppression that contributes to the severity of disease manifestation. In addition, IBRV is one of the bovine respiratory disease complex pathogens, which is the leading cause of cattle death around the world [2–4]. Taken together, it is a major disease of cattle leading to significant economic losses to the dairy industry worldwide [5, 6]. Several developed countries such as Germany, have adopted immunization and eradication of pathogen-positive animals for IBR control [7]. Therefore, early identification of IBRV positive animals is critical for disease control and elimination programs to diminish its burden on the dairy industry.

At present, IBRV can be routinely detected using cell culture, polymerase chain reaction (PCR), immune-histopathology, and enzyme-linked immunosorbent assay (ELISA) as well as the virus neutralization test [8–10]. However, currently available diagnostic tests remain laboratory-based and require sophisticated instruments operated by specially trained personnel. Although isothermal amplification techniques such as the loop-mediated isothermal amplification (LAMP) have been offered as a simple, rapid and alternative molecular pathogen diagnostic tool for point-of-care testing in the field [11, 12], the LAMP assay needs four to six primers, leading to longer amplicons and possibly more difficult to design in the case of highly variable viruses.

Recombinase polymerase amplification (RPA), is a novel isothermal alternative to PCR, which targets and amplifies DNA from clinical samples with high sensitivity and specificity [13]. The technology takes advantage of three major proteins, including recombinase proteins, single-strand binding proteins (SSB), and polymerases, and relies on two specific oligonucleotide primers and a probe, and can specifically amplify nucleic acid sequences ranging from trace levels to detectable amounts of product in an isothermal format in less than 30 min. RPA products can be detected by gel electrophoresis, probe-based fluorescence monitoring or lateral flow dipsticks depending on the specific primers and/or probe configuration. Although RPA technology has been widely used for detection of various pathogens since its initial development [14–19], to date, there is no RPA assay developed for IBRV detection.

In this study, RPA primer and probe combinations were designed, and screened to permit LFD-RPA detection of IBRV. The development of a combination of LFD-RPA detection for IBRV was described. Finally, the performance of the RPA assay on acute phase fever clinical samples was evaluated and the results compared with SYBR Green I real time PCR.

## Methods

### Viruses, cell and clinical specimens

IBRV/BoHV-1.1/BarthaNu/67 strain, bovine herpesvirus 5 (BoHV-5)/N569 strain, bovine viral diarrhea virus (BVDV)/NADL strain, bovine parainfluenza virus type 3 (BPIV-3)/BN-1 strain were preserved in our laboratory, and the viruses were cultured in the Madin-Darby Bovine Kidney (MDBK) cell using the Dulbecco’s Modified Eagle Medium (DMEM, HyClone, USA), containing antibiotics (1000 UI/L penicillin, 100 mg/L streptomycin) with 8% horse serum. Other bovine virus strains for cross reactivity testing used in this study were provided by full-length cloned cDNA: entire genome sequence of bovine respiratory syncytial virus (BRSV)/A51908strain, bovine ephemeral fever virus (BEFV)/(Gene Bank No: NC_002526.1), bovine enterovirus (BEV)/VG-5-27strain, bovine coronavirus (BcoV)/ENT strain (Gene Bank No:NC_003045.1), respectively. A total of 106 acute-phase high fever clinical specimens (24 fecal, 36 blood, 38 nasal swab and 8 tissue specimens) were collected from cattle farms suspected to be infected with IBRV in Shandong Province, China.

### Isolation of DNA/RNA, cDNA synthesis

IBRV and other herpesviruses DNA were extracted from 200 μL of infected cell culture supernatant using the UNIQ-10 viral DNA extraction kit (Sangon Biotech Co., Ltd., Shanghai, China). RNA from BVDV, BPIV-3 was prepared from 200 μL of infected cell culture supernatant using the QIAamp viral RNA minikit according to manufacturer’s instructions (Qiagen, Germany), respectively. The DNA/RNA was eluted in 50 μL of nuclease-free water. The extracted RNA was used as template for cDNA synthesis using the RevertAid™ First Strand cDNA Synthesis Kit (Fermentas, Canada). All templates were stored at −70 °C until further needed.

### Generation of DNA standard

The IBRV UL52 region fragment (300 bp) were synthesized by Jierui Biotech (Shanghai, China) and cloned into pEASY-T3 vector, designated as UL-T3-RPA. The UL-T3-RPA standard DNA was extracted with Plasmiad Mini kit (Tiangen, Beijing) and measured using a Nanodrop ND-1000 spectrophotometer (Thermo Scientific, Dreieich, Germany). The DNA copy number was calculated by the equation: DNA cope number = (M × 6.02 × 10^23^ × 10^−9^)/(n × 660)^28^, M: molecular weight, n: plasmid concentration measured at 260 nm. DNA standard was stored at −20 °C until further used.

### Design of RPA primers and specific LF probe for IBRV

The highly conserved region of IBRV was screened and selected in GenBank database (GenBank: AJ004801.1, E01200.1, NC_001847.1, JX898220.1, KM258880.1). Eight forward primers and reverse primers, and nine LF probes (Table 1) were designed according to RPA instruction manual from TwistDx (TwistDx, Ltd., Cambridge, United Kingdom). In summary, the length of 30 bp to 35 bp for RPA Primers is recommended. TwistAmp™ LF Probe oligonucleotide backbone includes a 5′-antigenic label FAM group, an internal abasic nucleotide analogue ‘dSpacer’ and a 3′-polymerase extension blocking group C3-spacer. One amplification primer opposing TwistAmp™ LF Probe is labeled with Biotin at its 5′ end, thus dual-labeled amplicon can be detected simultaneously. Oligonucleotide RPA primers and LF probes used in the study were synthesized by Sangon Biotech (Shanghai, China).

**Table 1:** The sequences of primers and probes designed in this study

Primers	Oligonucleotide sequences (5’to3’)	Product sizes (bp)	Location
1F:	5′-GGCAAGCGCCGCGAAGGAAACCAAGTCG-3′	236	4013–7237
1R:	【Biotin】 5′-GGCTTTACTCCAACAGCGGTGCGACTCCCTTC-3’		
1LF:	5′-【FAM】GCGAAGGAAACCAAGTCGGGGAAGGCGAGTC[dSpacer]CGAGGGGTTAGGCG【C3-spacer】-3’		
2F	5′- GGGGACGGCCACGTACACTTGCTCGTCGTAGAGG −3′	187	19,597–20,823
2R:	【Biotin】5′- TCTGTCCGACGGCGGCAACGGCAACCCAATC −3’		
2-1LF1:	AAAGGCGTTTCGCAGCGAGGCGCCAAAGGG[dSpacer]CTCAGCAGGGCGGAGAC-3′		
2-2LF2:	5′-【FAM】AGGCGTTTCGCAGCGAGGCGCCAAAGGGG[dSpacer]TCAGCAGGGCGGAGACGT【C3-spacer】-3’		
3F:	5′- ACACGAGGTCGCCGCCCGTGAAGACGCCGTTGTT −3′	142	23,526–25,889
3R	【Biotin】5′- CCAATGGACGGAAAGGTGCCCGCCTCCCAGAT −3’		
3LF:	5′-【FAM】CCGTTGTTGGTGGCCTTGCGGATCTTGCAG[dSpacer]AGTAGAGCCCCGTCTTA【C3-spacer】-3’		
4-1F:	5′- TTGACAAAGACCGAGTCCGTGTCGCCGTAAACCA −3′	274	45,238–48,978
4-1R:	【Biotin】5′- TCGATAAGCAGCAGGCCGCCATCAAGGTCGTGT −3′		
4-1LF:	5′-【FAM】TGTGGCCCAGCGCTCGTGTATGTACCGCCG[dSpacer]GTCTCGAGCAGCATGTC【C3-spacer】-3’		
4-2F	5′-GCTTTCCGACCAGCGCCGAATCTTGGATGCAGT −3′	250	47,151–47,397
4-2R:	【Biotin】5′- GGCCAAAACCGCTTTCAGAAGCAGAGCAAGGTGA −3’		
4-2LF:	5′-【FAM】CCGCAAAGTGCCGAGGGATGTCCTTGTAGT[dSpacer]CAGGTCCACCTTCCGCT【C3-spacer】-3’		
4-3F:	5′- GCCACCGCCGACAGCTCCAGGTGGGGCAAGTAC −3′	295	47,151–47,397
4-3R:	【Biotin】5′- CGGGCCAAAACCGCTTTCAGAAGCAGAGCAAGGT −3’		
4-3LF:	5′-【FAM】CACCTTCCGCTCCCGCAGGGCCTCCTCGGC[dSpacer]ACGGCGTTGAGCTTGTA【C3-spacer】-3′		
4-4F	:5′- CTTTCCGACCAGCGCCGAATCTTGGATGCAGTA −3′	154	47,151–47,397
4-4R:	【Biotin】5′- CAGCTACAAGCTCAACGCCGTGGCCGAGGAGGC −3’		
4-4LF:	5′-【FAM】CCGCAAAGTGCCGAGGGATGTCCTTGTAGT[dSpacer]CAGGTCCACCTTCCGCTC【C3-spacer】-3′		
gB-F:	5′- ACACGGACCGCGTGCCCGTGGGCATGGGCGAGATCA −3′	257	55,015–58,579
gB-R:	【Biotin】5′- GCAGTTCACAGAGGTGCCCGTGCGGTAGAGCC −3’		
gB-LF:	5′-【FAM】AGCGGGCGCAAGGTGGTGGCCTTTGACCGC[dSpacer]ACGACGACCCCTGGGAG【C3-spacer】-3′		

Note: *F*: Forward primer; *R*: Reverse primer; *LF* Probe; *FAM* Carboxyfluorescein; *dSpacer* A tetrahydrofuran residue; C3-spacer: 3′-block

### Laboratory based RPA assays

TwistAmp™ nfo kits were supplied as dry enzyme pellets in eight strips within vacuum-sealed pouches (TwistDx, Ltd., Cambridge, United Kingdom). The IBRV RPA was performed in a 50 μL final reaction volume according to the instructions outlined in the Twist Amp nfo kit manual. The rehydration solution contained an optimized blend of DNA template and primers and a LF probe. Each reconstituted reaction mixture contained 2 μL DNA template, 2.1 μL (10 μM) Primer, 0.6 μL (10 μM) TwistAmp™ LF Probe, 29.5 μL Rehydration Buffer. Resuspend the reaction pellet with 47.5 μL of the rehydration solution, and then the reaction was initiated by adding 2.5 μL of Magnesium Acetate (280 mM). The tubes were then incubated at 38 °C in an incubator block for 4 min. As recommended, the samples were blended up and down 6–8 times after 4 min’ incubation, and an additional incubation was continued for 21 min. In addition, in each run, a positive template control supplied with the Twist Amp nfo kit (primers/probe and template) and a negative template control (nuclease-free) water were included.

The amplicon of TwistAmp™ LF Probe system was visualised using a simple ‘sandwich’ LFD assay. Milenia’s Genline Hybridetect-1 or Hybridetect-2 lateral flow strips duplexes labeled with anti-FAM gold conjugates and anti-Biotin antibodies from Milenia GmbH (Germany) were used in this study for detection of LFD-RPA amplified nucleic acids. 2 μL of hybridization products were mixed with 98 μL of PBST (1 × Phosphate Buffered Saline with 0.1% Tween-20) running buffer. The LFD were then placed into the PBST dilution in a 96-well plate. The positive amplification product might be indicated by both test line and control line on the strip visualized simultaneously after 5 min, the negative control (no template) should generate a separate control line found further up test line on the strip. The absence of a control line on the LFD indicates the strip could not work correctly.

The outcome of TwistAmp™ LF Probe systems were also analyzed by agarose gel-electrophoresis (AGE). In brief, the amplification product was purified by commercial PCR purification kits. The comparable size of required amount of the amplification products were visualized by electrophoresis on a 2% agarose-gel.

### Determination of sensitivity and specificity of the assay

To determine the limit of IBRV genomic DNA copies, ten-fold serial dilutions of the standard UL-T3-RPA plasmid ranging from 5 × 10^8^ to 5 × 10^−1^ DNA copies per reaction were detected within the same sample run. The operation steps were performed according to section of laboratory based RPA assays. The assay sensitivity assessment was also carried out by PCR assay, and the IBRV DNA amplification condition were previously described [20]. Briefly, the nucleotide sequences of the primers were as follows: BF: 5′-AGACCCCAGTTGTGATGAATGC-3′ and BR: 5′-ACACGTCCAGCACGAACACC-3′. The amplification conditions consisted of a preliminary denaturation step of 95 °C for 4 min, 35 cycles of denaturation at 95 °C for 1 min, primer annealing at 58 °C for 45 s, and extension at 72 °C for 45 s and a final cycle of 72 °C for 5 min, and the size of amplification product is 183 bp. Two methods were operated with the same amount of template.

The specificity of the assay was assessed among other viral pathogens of cattle with similar clinical signs or other herpesviruses. DNA and RNA extracts were prepared from cell culture supernatant of BoHV-5, BVDV, BPIV-3, respectively, and cDNA of BVDV, BPIV-3 were prepared as mentioned above. Clone of full-length genome of BRSV, BEFV, BEV and BcoV were supplied as templates in the LFD-RPA reaction. Additionally, DNA extracted from 5 × 10^5^ DNA copies per reaction of IBRV and IBRV-free samples were used as a positive control and a negative control respectively.

### Clinical specimen preparation

A total of 106 acute-phase high fever clinical specimens including 24 fecal, 36 blood specimens, 38 nasal swabs and 8 tissue specimens were collected from in non-vaccinated herds suspected to be infected with IBRV in Shandong Province, China. Tissue specimens were homogenized in 10 mM phosphate buffered saline (PBS) with a dilution of 1:10 (*W*/*V*), centrifuged at 10,000×g at 4 °C for 15 min, sample suspension was collected until used. Fecal and swabs were placed immediately in 1 mL PBS and mixed by pulse-vortexing for 20 s after collection. 200 μL sample suspension was separated for DNA extraction using magnetic beads (Dynabeads®SILANE viral NA kit, Life Technologies, Darmstadt, Germany) according to the manufacturer’s instructions. In summary, 200 μL of sample suspension was incubated with 50 μL (20 mgmL^−1^) of proteinase K and 300 μL of lysis/binding buffer (viral NA) for 5 min at room temperature, and then 150 μL of isopropanol and 50 μL (2 mg) of Dynabeads were added and incubated at room temperature for 10 min followed by two washing steps. Viral DNA bound to the Dynabeads was left to dry at room temperature for 5 min before eluting in 50 μL of elution buffer. A volume of 2 μL of DNA extracted from each swab specimen was used as a template in the RPA reactions. The minute was how long before a test line was observed on the LFD.

### SYBR green I real-time PCR for amplification of IBRV

The primer pairs for SYBR Green I real-time PCR used for amplification of IBRV were IBRV-F: 5′- ACGGACGACGAGCTGGGACT-3′, IBRV-R: 5′-CGGCAGCGAAACCATGAAAT-3′. Real-time PCR was performed on a LightCycler480 Real-time PCR System using SYBR® Premix Ex Taq™ II (TliRNaseH Plus) reagents (Takara BioInc, Japan) according to the Manufacturer’s instructions. The reaction was carried out in a 20 μL volume containing 10 μL 2 × SYBR Premix Ex Taq II, 0.5 μL of forward and reverse primers (20 μM), respectively, 2 μL of DNA and 7 μL DNase-free water. The real-time PCR was performed as follows: 1 cycle of 95 °C for 5 min, followed by 45 cycles of 95 °C for 30 s and 63 °C for 30 s. Fluorescent signal was collected during the elongation step. In diagnostic real-time PCR assay, it was customary to regard results between C_T_ 35 and 40 as equivocal and above C_T_ 40 as negative.

## Results

### Design and optimization of RPA primers and probe

**Fig. 1:**
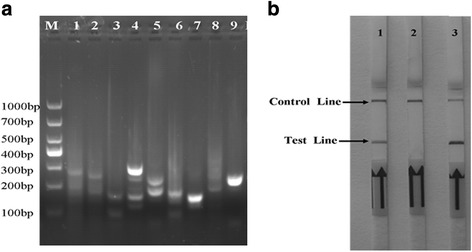
Validating the designed primers/probe of IBRV LFD-RPA assay. **a** Agarose gel electrophoresis of RPA products generated using designed primers/probes. Lane M: molecular weight standard (DNA Marker1000). Lane 1 to 9: designed primer and probe sets. Especially, Lane 5 was primers/probe set 4-2F/4-2R/4-2LF, and the expected size of the product was 247 bp. **b** Lateral-flow strip end-point analysis of RPA products generated using optimal designed primers/probes set 4F/4R/4LF. Lane 1: positive control (supplied by Twist Ampnfo kit); Lane 2: negative control (DNase-free water); Lane 3: IBRV amplicons performed with RPA primer pair 4F/4R/4LF. Samples were tested in triplicate with one reaction displayed in figure for each triplicate

Based on alignment analysis of infections bovine rhinotracheltis virus (GenBank: AJ004801.1, E01200.1, NC_001847.1, JX898220.1, KM258880.1), six distinct regions were selected as a candidate target for designing the LFD-RPA primers and probes. Eight forward primers and eight reverse primers labeled at the 5′ end with biotin for the detection of IBRV were designed to screen candidate primer pairs and probe (Table 1). Then nine LF probe combinations with primers were employed for further validation in this study. The candidate primers/probe for the LFD-RPA assay were screened by performing with TwistAmp™ nfo reactions and preliminarily analyzed on 2% agarose gel with labeled amplicons. The initial agarose gel result showed that Primer set 4-2F/4-2R/4-2LF yielded specific amplification efficiency for the RPA assay, and produced the expected size of the product was 250 base-pairs (Fig. 1a), while the primers/probe targeting glycoprotein gB of the IBRV genome in this study could not be used to amplify effectively in the initial screen (data not show). In order to determine the best pairs of primers/probe for maximum effective amplification, a series of staggered primers with 1–5 base intervals upstream and downstream of the original 4-2F/4-2R/4-2LF sequences were designed to obtain optimal primers for IBRV RPA, LFD-RPA results indicated that Primer set 4F/4R/4LF presented the most efficient amplification with expected size of 247 bp (Table 2), showing a test line within 5 min on the LFD (Fig. 1b).

**Table 2:** RPA primers and probe used for the rapid detection of IBRV

Name	Sequence (5’to 3′)
4F	5′-TTCCGACCAGCGCCGAATCTTGGATGCAGTACT-3’
4R	5′-【Biotin】GGCCAAAACCGCTTTCAGAAGCAGAGCAAGGTGA-3’
4LF	5′-【FAM】CCGCAAAGTGCCGAGGGATGTCCTTGTAGT【dSpacer】CAGGTCCACCTTCCGCT【C3-spacer】-3’

Note: *F* Forward primer; *R* Reverse primer; *LF* probe; *FAM* Carboxyfluorescein; *dSpacer* A tetrahydrofuran residue; C3-spacer: 3′-block

### Sensitivity and specificity of the RPA reaction

**Fig. 2:**
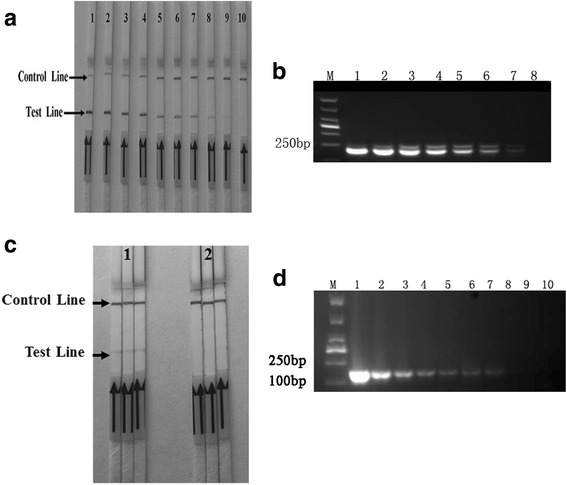
Sensitivity of the LFD-RPA assay. **a** Molecular sensitivity of RPA using total DNA extracted from 10-fold serially diluted IBRV-infected cell as template. IBRV templates of lane 1 to 10 in these reactions extracted from ten-fold serially diluted virus range from 5 × 10^8^ to 5 × 10^−1^ DNA copies per reaction. Samples were tested in triplicate with one reaction displayed in figure for each triplicate. **b** IBRV RPA reaction products (247 bp) could be detected on a 2% agarose gel. Lane 1 to 9 were represented IBRV RPA reactions extracted from ten-fold serially diluted IBRV templates range from 5 × 10^6^ to 5 × 10^−1^ DNA copies per reaction. **c** Repeatability of limits detection. The sensitivity of the assay using IBRV DNA extracted from 5 copies per reaction of IBRV DNA and negative control (DNase-free water) were also evaluated in 3 replicates, respectively. **d** Sensitivity of conventional PCR. Molecular sensitivity of conventional PCR uses the same template amount as the IBRV LFD-RPA assay. Templates of lane 1 to 10 in these reactions extracted from ten-fold serially diluted virus genome DNA range from 5 × 10^8^ to 5 × 10^−1^copies per reaction. Samples were tested in triplicate with one reaction displayed in figure for each triplicate

The analytical sensitivity of the RPA assay was determined using the IBRV DNA extracted from ten-fold serially diluted IBRV DNA ranging from 5 × 10^−1^ to 5 × 10^8^ copies per reaction, and the minimum virus detection limits of RPA were as low as 5 copies per reaction (Fig. 2a). The amplified products in the IBRV LFD RPA reaction were also detected by subsequent agarose gel electrophoresis (Fig. 2b). To detect the stability of the RPA method, the stability of the assay using DNA extracted from 5 × 10^0^ DNA copies per reaction of IBRV and negative controls were also evaluated in 3 replicates, respectively. The detection limits of the method showed the same result (Fig. 2c). As compared, all these ten-fold serially diluted templates were detected by conventional PCR, the sensitivity of the gold standard conventional PCR was 5 × 10^2^ DNA copies per reaction. Therefore, the sensitivity of our RPA-LFD assay was at least 100 times higher than that of the routine PCR assay (Fig. 2d).

**Fig. 3:**
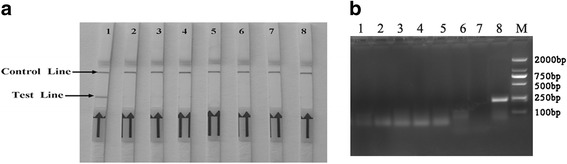
Specificity of the LFD-RPA assay. **a** The specificity of the IBRV RPA-LFD assay was assessed for other viral pathogens genome cDNA of cattle that present similarly in the clinic other herpesviruses such as bovine herpesvirus5 (BoHV-5). Lanes 1: positive control. Lanes 2 to 8: BVDV, BPIV-3, BRSV, BEFV, BEV, BcoV and BoHV-5, respectively. Samples were tested in triplicate with one reaction displayed in figure for each triplicate. **b** The specificity of the IBRV RPA-LFD products could be displayed on a 2% agarose gel. Lanes 1 to 7: BVDV, BPIV-3, BRSV, BEFV, BEV, BcoV and BoHV-5, respectively, Lane 8: positive control. Lane M: molecular weight standard (DNA Marker 2000)

The specificity of the assay was assessed with other viral pathogens of cattle with similar clinic and other herpesviruses such as bovine herpesvirus5 (BoHV-5).As shown in Fig. 3a, no cross-reactions of the BVDV, BPIV-3, BRSV, BEFV, BEV, BcoV, and BoHV-5 were observed and the amplicons in these viruses examined by the IBRV RPA-LFD assay were also tested with agarose gel electrophoresis Fig. 3b.

### RPA performances using clinical specimens

One hundred six clinical specimens were collected from suspected cases, and viral DNA extracted using magnetic beads were used as templates for detection. In all cases, clinical specimens were subjected to conventional PCR, RPA, and compared with the real-time PCR assay. The results of those assays showed that a total of 76 clinical specimens were tested positive by conventional PCR, while the same performance that 84 specimens were detected positive by IBRV RPA nucleic acid amplification assays on LFD within 5 min, and with the Cycle threshold (C_T_) values below 35 using the SYBR Green I real-time PCR assay. This particular RPA therefore was highly sensitive compared with the real-time PCR, and there was very good correlation between the results from RPA and SYBR Green I real-time PCR of the same sample (Table 3). In addition, the results show that IBRV RPA-LFD may be an ideal method to detect the IBRV genome in low resource settings.

**Table 3:** Comparative performance of LFD-RPA, PCR and SYBR Green I real time qPCR assays for detection of IBRV suspected clinical specimens

Samples name	PCR		LFD-RPA		Real-time qPCR		LFD-RPA/Real-time qPCR		LFD-RPA/ PCR/ Real-time qPCR	
	P	N	P	N	P	N	P	N	P	N
fecal	18	6	20	4	20	4	20	4	18	4
blood	30	6	32	4	32	4	32	4	30	4
nasal swab	20	18	24	14	24	14	24	14	20	14
lymph node	3	0	3	0	3	0	3	0	3	0
spleen	3	0	3	0	3	0	3	0	3	0
lung	2	0	2	0	2	0	2	0	2	0
Total	76	30	84	22	84	22	84	22	76	22

Note: *P* Positive; *N* Negative

## Discussion

Rapid and reliable nucleic acid tests are the gold-standard for the detection of a wide variety of infectious pathogens. Recent studies have shown that isothermal amplification techniques such as RPA and LAMP could be developed to amplify nucleic acids, which require neither high precision instrument for amplification nor elaborate methods for detection of the amplified products [21]. Among those isothermal amplification assays, RPA is a powerful, innovative nucleic acid test, which could be useful for low resource settings [22, 23]. RPA outperforms over LAMP currently developed for IBRV diagnosis. One advantage is that RPA reaction only utilizes two primers and one probe and three binding sites, in contrast, the LAMP assay needs at least four primers and six binding sites. In addition, RPA reagents provided in a lyophilized pellet are stable even when stored for 3 weeks at 45 °C, which allow independence from the cooling chain [24, 25].

The most important step of RPA is to identify the appropriate primers and probes. However, there is no ideal design support software available for RPA. In this study, different conserved genomic regions were selected for olignucleotide design (Table 1). Several primer and probe combinations were initially evaluated for target sequences and two products generated by RPA primer pair and probe were distinct in size on an agarose gel to exclude non-specific amplification (Fig. 1a). It is worth mentioning that, unlike PCR-based DNA amplification assays, it should be noted that the primer and probe set of RPA is much more stringent as it requires longer primers (30–35 bases) and probes (46–52 bases) leading to the potential of formation of secondary structures. Moreover, after several optimization screening, one optimal set of the primers and probe limited to the UL52 regions functioning DNA replication can be successfully used to amplify an exact sequence-matched template (Fig. 1b) since replacement, increase, decrease or mismatch of single base will have a significant impact on the result of amplification [26]. Furthermore, we also found that IBRV molecular detection with RPA assay poses unique challenges because the genome of IBRV was rich in GC content. In retrospect, given recently published data describing glycoprotein-based IBRV detection assay, loop-mediated isothermal amplification (LAMP) or PCR-based assays have been reported to detect IBRV in biological samples [27, 28]. In the present study, none of the primers targeting glycoprotein gB within the same region of the genome of LAMP and PCR could be used to amplify effectively in the initial screen (Table 1), this may be due to the long probe formed folded secondary structures and had an impact upon hybridization to target, or perhaps there are different regions for primer design between RPA and LAMP/PCR.

With regard to analytical sensitivity, the RPA developed in this study showed a performance limit of detection of 5 copies per reaction of IBRV DNA (Fig. 2a and b) equaled to Real Time PCR, which was at least 100 times higher than that of the routine PCR assay (Fig. 2d). The study of specificity showed that no cross-detection between IBRV and other bovine infectious virus or bovine herpesvirus 5 (Fig. 3a and b). It should be noted that the high sensitivity and specificity of this assay gave the credit to good use of gold-labeled anti-FAM and anti-Biotin antibodies to capture RPA product simultaneously. The clinical performance of PCR, LFD-RPA and SYBR Green I based PCR assays were detected using 106 suspected clinical specimens, the clinical sensitivity of both LFD-RPA and SYBR Green I real-time assays was 100%, while PCR showing the lower detection might be due to some clinical samples containing low viral titers. Although LFD-RPA is not appropriate for the quantitative analysis of nucleic acid, an important feature of the method is the LFD-RPA assay under normal circumstances outperforms other isothermal amplification assays in terms of simplicity and sensitivity, because it only requires a simple heat block to run and the results can also be visualized by LFD with naked eyes inspection.

This methodology of LFD-RPA might be uniquely appropriate for application of visual field testing due wholly to the RPA enzymes can be short-term storage and transport at ambient temperatures in a lyophilized pellet, and conducted without a precise incubation temperature (body temperature) [29]. Moreover, some samples only require simple preparation and processing before RPA. It has also been shown that RPA tolerates multiple known PCR inhibitors, including hemoglobin (50 gL^−1^), heparin (0.5 U), undiluted serum, and ethanol (4%*v*/v) [17]. However, some components of nucleic acids extracted from clinical sample have an influence on RPA reaction such as the concentrations of background DNA in whole blood [30]. To fulfill the full potential of developed IBRV LFD-RPA at the point of care in field conditions, we evaluated several simple methods for clinical sample extraction including direct sample lysis with heat lysis, alkaline lysis (0.2 M KOH or 0.5% SDS) and Dynabeads® magnetic separation. We found that Dynabeads® magnetic separation worked well. Similar sensitivities were obtained from viral genome extraction kit. Heat and alkaline lysis did not result insufficient recovery of positive DNA (data not shown). Although Dynabeads® magnetic separation technology is easily adapted to automated liquid handling platforms, other methodology platform to perform sample preparation will be further required to optimize.

## Conclusion

This workpackage has developed a rapid, robust, cost-effective and highly sensitive LFD-RPA assay for the detection of IBRV. In addition, the rapid LFD-RPA for IBRV may save much time in epidemiological surveillance and aid in identification of latent IBRV infected individuals in beef and dairy farms. Furthermore, it may be serves as a model platform for detecting other bovine pathogens.

## Abbreviations

BcoV: Bovine coronavirus
BEFV: Bovine ephemeral fever virus
BEV: Bovine enterovirus
BoHV-1: Bovine herpesvirus 1
BPIV-3: Bovine parainfluenza virus type 3
BRSV: Bovine respiratory syncytial virus
BVDV: Bovine viral diarrhea virus
DMEM: Dulbecco’s modified eagle medium
ELISA: Enzyme-linked immunosorbent assay
IBRV: Infectious bovine rhinotracheitis virus
LAMP: Loop-mediated isothermal amplification
LFD: Lateral flow dipstrip
MDBK: Madin-darby bovine kidney
PBS: Phosphate buffered saline
PCR: Polymerase chain reaction
RPA: Recombinase polymerase amplification
SSB: Single-strand binding proteins

## References

[1] Straub OC (2001). Advances in BHV1 (IBR) research. Dtsch Tierarztl Wochenschr.

[2] Kirchhoff J, Uhlenbruck S, Goris K, Keil GM, Herrler G (2014). Three viruses of the bovine respiratory disease complex apply different strategies to initiate infection. Vet Res.

[3] Muylkens B, Thiry J, Kirten P, Schynts F, Thiry E (2007). Bovine herpesvirus 1 infection and infectious bovine rhinotracheitis. Vet Res.

[4] Nandi S, Kumar M, Manohar M, Chauhan RS (2009). Bovine herpes virus infections in cattle. Anim Health Res Rev.

[5] Raaperi K, Orro T, Viltrop A (2014). Epidemiology and control of bovine herpesvirus 1 infection in Europe. Vet J.

[6] Newcomer BW, Givens D (2016). Diagnosis and control of viral diseases of reproductive importance: infectious bovine Rhinotracheitis and bovine viral diarrhea. Vet Clin North Am Food Anim Pract.

[7] Nardelli S, Farina G, Lucchini R, Valorz C, Moresco A, Dal Zotto R, Costanzi C (2008). Dynamics of infection and immunity in a dairy cattle population undergoing an eradication programme for infectious bovine Rhinotracheitis (IBR). Prev Vet Med.

[8] Biswas S, Bandyopadhyay S, Dimri U, Patra PH (2013). Bovine herpesvirus-1 (BHV-1) – a re-emerging concern in livestock: a revisit to its biology, epidemiology, diagnosis, and prophylaxis. Vet Q.

[9] Graham DA, Mawhinney KA, McShane J, Connor TJ, Adair BM, Merza M (1997). Standardization of enzyme-linked immunosorbent assays (ELISAs) for quantitative estimation of antibodies specific for infectious bovine rhinotracheitis virus, respiratory syncytial virus, parainfluenza-3 virus, and bovine viral diarrhea virus. J Vet Diagn Invest.

[10] Yang SH, Wang CF, Gao YD, Liu WH, Yan-Qin LI, Zhong JF (2007). Fast Diagnosis of IBRV by Nested-PCR Method. Acta Ecologiae Animalis Domastici.

[11] Fu S, Qu G, Guo S, Ma L, Zhang N, Zhang S, Gao S, Shen Z (2011). Applications of loop-mediated isothermal DNA amplification. Appl Biochem Biotechnol.

[12] Suwancharoen D, Sittiwicheanwong B, Wiratsudakul A (2016). Evaluation of loop-mediated isothermal amplification method (LAMP) for pathogenic Leptospira spp. detection with leptospires isolation and real-time PCR. J Vet Med Sci.

[13] Piepenburg O, Williams CH, Stemple DL, Armes NA (2006). DNA detection using recombination proteins. PLoS Biol.

[14] Boyle DS, Lehman DA, Lillis L, Peterson D, Singhal M, Armes N, Parker M, Piepenburg O, Overbaugh J (2013). Rapid detection of HIV-1 Proviral DNA for early infant diagnosis using recombinase polymerase amplification. MBio.

[15] Euler M, Wang Y, Nentwich O, Piepenburg O, Hufert FT, Weidmann M (2012). Recombinase polymerase amplification assay for rapid detection of Rift Valley fever virus. J Clin Virol.

[16] Euler M, Wang Y, Heidenreich D, Patel P, Strohmeier O, Hakenberg S, Niedrig M, Hufert FT, Weidmann M (2013). Development of a panel of recombinase polymerase amplification assays for detection of biothreat agents. J Clin Microbiol.

[17] Kersting S, Rausch V, Bier FF, Nickisch-Rosenegk v (2014). M: rapid detection of plasmodium falciparum with isothermal recombinase polymerase amplification and lateral flow analysis. Malar J.

[18] Murinda SE, Ibekwe AM, Zulkaffly S, Cruz A, Park S, Razak N, Paudzai FM, Ab Samad L, Baquir K, Muthaiyah K (2014). Real-time isothermal detection of Shiga toxin-producing Escherichia Coli using recombinase polymerase amplification. Foodborne Pathog Dis.

[19] Xia X, Yu Y, Hu L, Weidmann M, Pan Y, Yan S, Wang Y (2015). Rapid detection of infectious hypodermal and hematopoietic necrosis virus (IHHNV) by real-time, isothermal recombinase polymerase amplification assay. Arch Virol.

[20] Yason CV, Harris LM, Mckenna PK, Wadowska D, Kibenge FS (1995). Establishment of conditions for the detection of bovine herpesvirus-1 by polymerase chain reaction using primers in the thymidine kinase region. Can J Vet Res.

[21] Niemz A, Ferguson TM, Boyle DS (2011). Point-of-care nucleic acid testing for infectious diseases. Trends Biotechnol.

[22] Zaghloul H, El-Shahat M (2014). Recombinase polymerase amplification as a promising tool in hepatitis C virus diagnosis. World J Hepatol.

[23] James A, Macdonald J (2015). Recombinase polymerase amplification: emergence as a critical molecular technology for rapid, low-resource diagnostics. Expert Rev Mol Diagn.

[24] Notomi T, Okayama H, Masubuchi H, Yonekawa T, Watanabe K, Amino N, Hase T (2000). Loop-mediated isothermal amplification of DNA. Nucleic Acids Res.

[25] Lillis L, Siverson J, Lee A, Cantera J, Parker M, Piepenburg O, Lehman DA, Boyle DS (2016). Factors influencing recombinase polymerase amplification (RPA) assay outcomes at point of care. Mol Cell Probes.

[26] Daher RK, Stewart G, Boissinot M, Boudreau DK, Bergeron MG (2015). Influence of sequence mismatches on the specificity of recombinase polymerase amplification technology. Mol Cell Probes.

[27] El-Kholy AA, Abdelrahman K, Soliman H (2014). Rapid detection of BoHV-1 genomic DNA by loop-mediated isothermal amplification assay. J Virol Methods.

[28] Pawar SS, Meshram CD, Singh NK, Sonwane AA, Saini M, Rautmare SS, Muglikar DM, Mishra BP, Gupta PK (2014). Rapid detection of bovine herpesvirus 1 in bovine semen by loop-mediated isothermal amplification (LAMP) assay. Arch Virol.

[29] Crannell ZA, Rohrman B, Richards-Kortum R (2014). Equipment-free incubation of recombinase polymerase amplification reactions using body heat. PLoS One.

[30] Rohrman B, Richards-Kortum R (2015). Inhibition of recombinase polymerase amplification by background DNA: a lateral flow-based method for enriching target DNA. Anal Chem.

